# A novel brachytherapy and chemotherapy integrated ureteral stent: In vitro and in vivo study

**DOI:** 10.1002/btm2.70077

**Published:** 2025-09-19

**Authors:** Xiaotian Yang, Xueliang Zhou, Zhanyun Zhou, Yipu Li, Chengzhi Zhang, Yingqi Liu, Xiaohan Ma, Yanan Li, Yebin Wang, Dechao Jiao

**Affiliations:** ^1^ Department of Clinical Medicine Zhengzhou University Zhengzhou China; ^2^ Department of Interventional Radiology The First Affiliated Hospital of Zhengzhou University Zhengzhou China

**Keywords:** ^125^Iodine seed, brachytherapy, chemoradiotherapy, doxorubicin, ureteral stent, urinary system

## Abstract

Ureteral carcinoma remains a major clinical challenge and requires effective localized treatment. Here, we report a novel ^125^I seed brachytherapy (ISB) and doxorubicin (DOX) chemotherapy integrated ureteral stent (IUS), which enables simultaneous urinary drainage and chemoradiotherapy. This study was divided into three parts. First, ISB and DOX significantly reduced T24 cell viability and inhibited migration and invasion in an in vivo study (*p* < 0.01). Second, a T24 xenograft mouse model demonstrated that the (DOX + ISB) group exhibited greater tumor suppression than the DOX (*p* = 0.08) and ISB (*p* = 0.02) groups, with decreased Ki‐67 and Bcl‐2 expression and increased apoptosis (all *p* < 0.01) in an in vitro study. Third, the IUS was successfully implanted in normal beagle dogs (*n* = 30) without surgical complications. The ureteral diameter increased with increasing cumulative brachytherapy and sustained DOX release (*p* < 0.05). Histological analysis revealed progressive tissue damage and fibrosis, with increased expression of α‐SMA, Caspase‐3, and Collagen‐1 in the 0.8 mCi + 20 mg DOX group (*p* < 0.05), whereas PCNA expression was highest in the Control group (0 mCi + 0 mg DOX). In conclusion, the newly designed IUS is safe and technically feasible in animals; clinical studies will be required to evaluate its use in humans.

**Translational Impact Statement**

This study presents an innovative integrated ureteral stent (IUS) that integrates urinary drainage with brachytherapy and chemotherapy. Two particularly important aspects of this work are as follows: (1) IUS has three functions: urine drainage, ^125^I seed brachytherapy, and localized doxorubicin chemotherapy; such multiple functions represent the development direction of future ureteral stents. (2) The combination of brachytherapy and chemotherapy has a distinct synergistic effect in vivo and in vitro studies. Moreover, the localized sustained release of DOX is lower than the concentration used in clinical intravesical instillation, effectively mitigating the cardiotoxicity. (3) The development of PLGA‐DOX drug delivery system provides a theoretical basis for the drug delivery of other drugs, such as immune drugs and targeted drugs, and can lay a better foundation for the drug delivery of other anticancer drugs in the future.

## 1 INTRODUCTION

Ureteral cancer (UC) is a malignant tumor of the urinary system with a poor prognosis and a high mortality rate of approximately 24.6%.^1^ The standard treatment for UC is nephroureterectomy with partial cystectomy; however, this operation is not safe for high‐risk patients such as elderly patients, patients with anesthesia contraindications, or patients' subjective refusal, who often can receive only palliative treatment with ureteral D–J stent implantation to relieve the high pressure of the collecting system.^2^, ^3^ However, such ureteral D–J stents have no anti‐cancer therapeutic effect,^4^ and developing a stent with multiple functions of urine drainage and anti‐cancer treatment is one of the future development directions for functional ureteral stents.

Polyurethanes (PUs) are synthetic polymers with good biocompatibility, low cytotoxicity, and good mechanical properties that can be used for delivering drugs.^5^ Previous studies have succeeded in producing nanomicelles of multiblock polyurethane with high loading capacity and encapsulation efficiency for doxorubicin (DOX)^6^; these nanomicelles could also be used in ureteral stents to deliver DOX.^7^, ^8^ Radioactive ^125^I seed brachytherapy (ISB) for the treatment of malignant solid tumors has been widely adopted in clinical practice and has demonstrated promising therapeutic outcomes.^9^ Our previous study demonstrated that ^125^I brachytherapy ureteral stents exhibit good safety and feasibility^10^; in an animal study, radioactive ISB can slowly and continuously release gamma rays, while nanomaterials loaded with DOX can also achieve slow drug release. This brachytherapy and chemotherapy mode can theoretically exert a synergistic effect.

Therefore, a novel ureteral stent capable of delivering both gamma rays and DOX was developed under the above assumption. This study aimed to assess the feasibility and safety of a ^125^I‐ and DOX‐integrated ureteral stent (IUS) through in vitro cell experiments and in vivo animal studies. Our research findings will offer valuable insights into the treatment of ureteral cancer and demonstrate significant potential for future clinical applications.

## 2 MATERIALS AND METHODS

### 2.1 Development of the IUS

#### 2.1.1 IUS

A newly designed ISB and DOX integrated ureteral stent measuring 20 cm in length and 6 French in diameter, featuring a double‐J structure at both ends (Tuoren Co. Ltd., Henan, China), was used. The IUS was designed with two separate cavities, a drainage and ^125^I seed cavity, which are isolated from each other. The cavities include an opening 5 cm from the proximal end that serves as a channel for ^125^I seed implantation. Each IUS is capable of holding ten ^125^I seeds, with the non‐seed section threaded with a 0.018‐inch guidewire (Cook Inc., Bloomington, IN). The ^125^I seeds (Saide Biological Technology Co., Ltd., Tianjin, China) measured 0.8 mm × 4.8 mm (diameter × length) and had a half‐life of 59.6 days. They emitted γ‐rays (35.5 keV) and X‐rays (27.4–31.5 keV) with an initial dose rate of 7.7 cGy/h and an effective irradiation distance of 17 mm. The DOX coating extended approximately 8 cm, covering the segment containing the radioactive ^125^I seeds.

#### 2.1.2 Drug loading process for the IUS

A total of 380 mg of PLGA (75:25, 110 kDa, Jinan Daigang Biomaterial Co., Ltd.) and 870 mg of DOX hydrochloride (98%, Macklin) were separately dissolved in 3.5 mL of dimethyl sulfoxide (DMSO, >99.5%, Sinopharm Chemical Reagent Co., Ltd.) and 7.0 mL of tetrahydrofuran (THF, ≥99.5%). The two solutions were then combined at a 1:2 (v/v) ratio to prepare the DOX‐containing coating solution.

The stent was mounted on an ultrasonic spraying device (MediCoat BCC‐300, SonoTek Corporation, Milton, NY) (Figure S5, Supporting Information). For the first group, the infusion rate was set to 0.04 mL/min, the spray length was 100 mm, the movement speed was 0.8 mm/min, and the number of spray cycles was from 5 to 6. For the second group, the infusion rate was adjusted to 0.03 mL/min, the spray length was 60 mm, the movement speed was 0.8 mm/min, and the number of spray cycles was from 3 to 4.

After spraying, the coated stents were placed in a forced‐air drying oven (DHG‐9053A, Shanghai Qixin Scientific Instrument Co., Ltd.) and dried at 40°C for 72 h. The dried stents were then subjected to irradiation sterilization at a dose of 15 kGy.

#### 2.1.3 Drug release in vitro

A total of 5.00 mg of DOX hydrochloride was accurately weighed into a small beaker and dissolved in 10 mL of normal saline, and the solution was then transferred to a 100‐mL volumetric flask. A standard solution was prepared with normal saline with a concentration of 500 μg/mL. Aliquots of 0.02, 0.10, 0.20, 0.40, 1.00, 2.00, 2.50, 3.00, 4.00, 5.00, 6.00, and 8.00 mL of the standard solution were each diluted to 10 mL with normal saline to obtain a series of working concentrations. The absorbance (A) of these solutions was measured at a wavelength of 485.0 nm, and normal saline was used as the blank control. A calibration curve was plotted, yielding the regression equation A = 0.007716c + 0.06638, with a correlation coefficient of *R* ^2^ = 0.9981 (Figure S1).

The samples were placed in physiological saline and incubated at 37°C in a constant‐temperature shaker. At 1, 3, 7, 14, and 28 days, samples were taken, and drug release was measured, with physiological saline used as the blank control.

### 2.2 Development of PU‐DOX membranes

#### 2.2.1 Preparation of PU samples

The drug‐loaded membrane was fabricated from the same material as the ureteral stent, which allowed for a better simulation of the product's effects in vivo. Thirty grams of polyurethane granules (2095A, Lubrizol) were weighed and compressed using a tablet press (Dongguan Xihua Testing Instrument Co., Ltd., XH‐406BEW‐30‐300) to obtain square polyurethane films with a thickness of 0.45 mm. These films were cut into rectangular samples of 1.5 × 5 cm. The cut samples were ultrasonically cleaned in anhydrous ethanol for 10 min and then removed and allowed to dry for later use.

#### 2.2.2 Preparation of the coating solution

PLGA (2.9 g, 50:50, Mw 110,000, Jinan Daigang Biotechnology Co., Ltd.) and 0.325 g of DOX hydrochloride (98%, Macklin) were dissolved separately in 51 g of a solvent mixture (DMSO [>99.8%, GC Aladdin] and THF [>99.5%, GC Macklin] in a 1.05:1 w/w ratio) to obtain a coating solution containing DOX.

#### 2.2.3 Preparation of the PU‐DOX membranes

The cleaned polyurethane samples 1.5 × 5 cm in the coating solution were immersed for 10 s and then transferred to a 70°C oven for 2 h. The immersion and drying process was repeated 4 times. Finally, the coated samples were cut into 0.8 × 0.8 cm squares, packaged, and subjected to irradiation sterilization at a dose of 15 kGy. The membrane thickness was measured at 0.05 cm, and the thickness of the DOX‐loaded layer was 0.05 μm.

#### 2.2.4 Drug release in vitro

The samples were placed in physiological saline and incubated at 37°C in a constant‐temperature shaker. At 1 h and 1, 3, and 5 days, samples were taken, and drug release was measured, with physiological saline used as the blank control.

### 2.3 Cell lines and in vitro experiments

#### 2.3.1 Cell lines

The human bladder transitional cell line T24 was obtained from the Cell Bank of the Chinese Academy of Sciences. The cells were cultured in Dulbecco's modified Eagle's medium (DMEM) supplemented with 10% fetal bovine serum (FBS, Gibco) and 1% penicillin/streptomycin (Meilunbio) at 37°C in a 5% CO_2_ atmosphere.

#### 2.3.2 CCK8 assays

T24 cells, either normally cultured or exposed to irradiation using an in vitro ^125^I radiation model (Figure S2) for 72 h, were seeded into 96‐well plates at a density of 5 × 10^3^ cells per well. Following treatment with different concentrations of DOX (0.04, 0.08, 0.12, 0.16, and 0.20 μg/mL), 100 μL of McCoy's 5A medium containing 10 μL of CCK‐8 reagent (Meilunbio, Han Bio) was added to each well at five distinct time points. The plates were incubated in the dark at 37°C for 4 h, and the absorbance was measured at a wavelength of 450 nm. Each group included six replicate wells, and the experiments were conducted in triplicate.

#### 2.3.3 Transwell migration and invasion assays

Transwell migration assays were performed using 24‐well Transwell chambers with an 8‐μm pore size (Corning). For invasion assays, the upper chambers were precoated with Matrigel matrix (BD Biosciences) to simulate the extracellular matrix. T24 cells, either normally cultured or subjected to 72 h of in vitro irradiation with ^125^I seeds, were seeded at a density of approximately 3 × 10^4^ cells per well into the upper chambers containing 200 μL of McCoy's 5A medium. The lower chambers were filled with 600 μL of McCoy's 5A supplemented with 10% FBS and DOX (1:1 volume ratio) at various concentrations (0.5, 1.0, 2.5, and 5.0 μg/mL). The plates were then incubated at 37°C for 24 h to allow for cell migration or invasion. Nonmigratory or noninvasive cells on the upper surface of the membrane were carefully removed using wet swabs. The cells that traversed the membrane were fixed with 4% paraformaldehyde, stained with crystal violet, and quantified under a microscope to assess their migratory and invasive capacities.

#### 2.3.4 Apoptosis assay

T24 cells were seeded in 6‐well culture plates at a density of 1 × 10^5^ cells per well and incubated overnight. The cells were divided into two groups: one cultured under normal conditions and the other subjected to brachytherapy with ^125^I seeds. Both groups were exposed to a gradient of DOX concentrations (0, 0.05, and 0.20 μmol/L). After 36 h of incubation, the cells were harvested, stained with Annexin V‐FITC/PI for 20 min at 4°C in the dark, and analyzed using a flow cytometer (BD FACSAria™ III, USA) for fluorescence quantification.

### 2.4 Mouse model and in vivo experiment

#### 2.4.1 Animal model and tumor volume

BALB/c‐nude mice (male, 4–8 weeks of age) were purchased from Beijing Huafukang Bioscience Co. Ltd. and maintained in microisolator cages under specific pathogen‐free (SPF) conditions at the Institutional Animal Care and Use Committee of Zhengzhou University (Approval number: ZZU‐LAC20250228[10]).

T24 tumor cells at 70%–80% confluence were trypsinized, centrifuged (5 min at 320*g*), and resuspended in PBS at a concentration of 1 × 10^7^ cells/mL. BALB/c‐nude mice were inoculated subcutaneously with T24 cells (5 × 10^6^ cells/mouse) in the right flank. When the tumor diameter reached 5–7 mm, BALB/c‐nude mice were randomly assigned to five groups: (1) Control, (2) PU, (3) DOX, (4) ISB, and (5) DOX + ISB (*n* = 5 per group). In the DOX and DOX + ISB groups, the PU‐DOX membrane was surgically applied to the surface of the tumor. Under fluoroscopy guidance (Siemens Artis Zeego DSA machine), an 18G puncture needle was inserted into the center of the tumor, and one ^125^I seed was implanted (using Siemens virtual navigation when necessary) (Figure 5a).

These groups were used to assess the effects of different treatment modalities and their impacts on the experimental outcomes. The tumor volume (V) was calculated using longitudinal cross sections (a) and transverse sections (b) as follows:

$$V=\frac{ab^{2}}{2}.$$

Animals were sacrificed for ethical reasons if the tumor reached 2000 mm^3^ in volume or became ulcerated.

#### 2.4.2 HE staining

Tumors, along with the heart, liver, spleen, lungs, and kidneys of the nude mice, were fixed in 4% formaldehyde, embedded in paraffin, and sectioned into 4 μm thick slices, which were mounted onto glass slides. The sections were deparaffinized using xylene and graded ethanol solutions (100%, 95%, 85%, 75%), followed by rinsing with distilled water. Hematoxylin and eosin (H&E) staining was performed, and the sections were dehydrated with increasing concentrations of ethanol and xylene. Finally, the sections were dried and examined under a microscope at 100× magnification.

#### 2.4.3 Immunohistochemistry

After the tumors were excised from the nude mice, the samples were embedded in paraffin blocks for Ki‐67 and Bcl‐2 detection. Protein expression levels in the tissue and cells were observed. The optical density values, which were calculated using ImageJ Pro software, served as an indirect measure of protein expression. These values were then compared statistically to evaluate the differences in expression.

#### 2.4.4 TUNEL staining

The tumor tissues from the nude mice were fixed with 4% paraformaldehyde (PFA), followed by two washes with PBS solution. The tissues were then incubated with the TUNEL reaction mixture for 60 min at 37°C in the dark. Afterwards, the cell nuclei were stained with DAPI for 10 min. TUNEL‐positive cells were observed using a confocal microscope (FV3000, Olympus, Japan), and the results were quantified using ImageJ software (USA).

### 2.5 Beagle dog experimentation

#### 2.5.1 Implantation of the IUS

The animal study was approved by the Institutional Animal Care and Use Committee of Zhengzhou University. Thirty healthy beagle dogs (15 males and 15 females weighing 15–16 kg) were randomly assigned to three groups (Group A: IUS loaded with 0.8 mCi ^125^I and 20 mg DOX; Group B: IUS loaded with 0.4 mCi ^125^I and 10 mg DOX; Group C: IUS loaded with 0 mCi ^125^I and 0 mg DOX; 10 dogs per group). The contralateral ureter served as the control. After successful anesthesia (intravenous injection of 3% pentobarbital sodium at a dose of 30–35 mg/kg), each animal was placed in the supine position, and the limbs were secured for skin preparation and disinfection of the lower abdomen. A midline incision was made in the lower abdomen, and a 2‐cm longitudinal incision was made in the bladder wall. Both ureters were carefully separated. Under fluoroscopy guidance (Artis Zeego, Siemens, Germany), a 7F catheter (Boston Scientific, Natick, MA) was retrogradely introduced into the ureter and renal pelvis with the aid of a guidewire. The catheter was then replaced with a newly designed IUS, ensuring that both stent ends were positioned in the renal pelvis and bladder. The contralateral ureter remained intact and served as the control. If the bladder incision was too large, it was covered with omentum and sutured. All wounds healed without complications. In accordance with the study protocol, three dogs were randomly euthanized by air embolism, and routine tests were conducted to assess blood parameters, liver function, renal function, and tissue reactions (Figure 6a).

#### 2.5.2 Serum biochemical examination

Blood samples (3 mL) were collected from the forelimb radial veins of beagle dogs at 1, 2, 4, and 8 weeks before the modeling procedure. Approximately 1 mL of an anticoagulant was added to each sample. Routine blood tests were then performed using an automated hematology analyzer. The remaining blood was transferred into a 5‐mL centrifuge tube and placed in a 37°C water bath for 15 min. The samples were subsequently centrifuged at 3000 rpm for 15 min. Biochemical tests, including routine blood analysis and liver and kidney function assessments, were conducted using a fully automated biochemical analyzer.

#### 2.5.3 Gross observation

In accordance with the experimental protocol, the kidney, ureter, and bladder were carefully isolated, and images were captured at 2, 4, and 8 weeks. The renal pelvis and upper ureter were longitudinally incised to assess the presence of hydronephrosis and to inspect the internal surface of the ureter for signs of bleeding, perforation, rupture, or ulceration.

#### 2.5.4 HE and Sirius red staining

Cross‐sectional samples from the IUS region were collected and fixed in formalin for subsequent hematoxylin and eosin (HE) staining at 2, 4, and 8 weeks following the initiation of the experiment. Histological analysis was conducted to assess submucosal infiltration by inflammatory cells (lymphocytes and neutrophils), epithelial layer thickness, collagen deposition, granulation tissue proliferation, and ureteral wall fibrosis according to established histological scoring criteria. Sirius red staining was employed to further evaluate the extent of fibrosis.

#### 2.5.5 Brachytherapy dosimetry

The tissue‐absorbed radiation doses at 5 and 10 mm transverse from the IUS at different time points in the 0.4 and 0.8 mCi groups were calculated using the following equation^11^:

$$\mathrm{D\gamma}=34.6\left(\sum \Delta \mathrm{i\phi i}\right)C_{0}T_{\mathrm{eff}}\left[1-e^{\frac{-0.693}{T_{\mathrm{eff}}}t}\right]\Delta i.$$

Because iodine‐125 seeds emit gamma rays, the absorbed dose can be calculated using the following formula:

$$\mathrm{D\gamma}=34.6\left(\Delta \mathrm{i\phi i}\right)C_{0}T_{\mathrm{eff}}\left[1-e^{\frac{-0.693}{T_{\mathrm{eff}}}t}\right],$$

where Δ*i* represents the constant absorbed dose, ϕ*i* is the energy fraction absorbed by the target (for iodine‐125 seeds, this value is 0.219), *C*_0_ is the radiation dose in tissues at *t* = 0 μCI/g = 0, and *T*_eff_ is the physical half‐life of iodine‐125 seeds.

#### 2.5.6 Immunohistochemistry

Cross‐sectional samples from the IUS region were harvested, embedded in paraffin, and analyzed via immunohistochemistry to assess the expression levels of key proteins, including PCNA, collagen, caspase‐3, and α‐SMA, at 2, 4, and 8 weeks post‐experiment initiation. The quantitative evaluation of protein expression was performed by measuring the optical density with ImageJ Pro software, providing an indirect yet robust assessment. The resulting statistical comparisons offered direct insights into differential protein expression patterns across the experimental timeline.

#### 2.5.7 Immunofluorescence staining

Immunofluorescence staining was performed on 5‐μm‐thick tissue sections. The sections were incubated overnight at 4°C with a 1:100 dilution of primary antibodies against collagen‐1, caspase‐3, α‐SMA, or PCNA (Sigma, USA). After three washes with PBS, the primary antibodies were detected using a 1:200 dilution of Cy3‐labeled secondary antibodies (Beyotime, China) for 1 h. Following another three washes with PBS, the slides were counterstained with DAPI (Beyotime, China) and visualized under a fluorescence microscope (Olympus BX53, Japan) at 200× magnification.

### 2.6 Statistical analysis

Quantitative data analysis was conducted using GraphPad Prism (version 10.4.1; GraphPad Software, LLC, USA). A t test was used to compare normally distributed data between two groups, and one‐way ANOVA was applied for comparisons across all groups. Pairwise comparisons within and between groups were conducted using the least significant difference (LSD) method. For rate comparisons, the Mann–Whitney *U* test was employed. A *p*‐value of less than 0.05 was considered statistically significant.

## 3 RESULTS

### 3.1 Design and characteristics of the IUS

**FIGURE 1:**
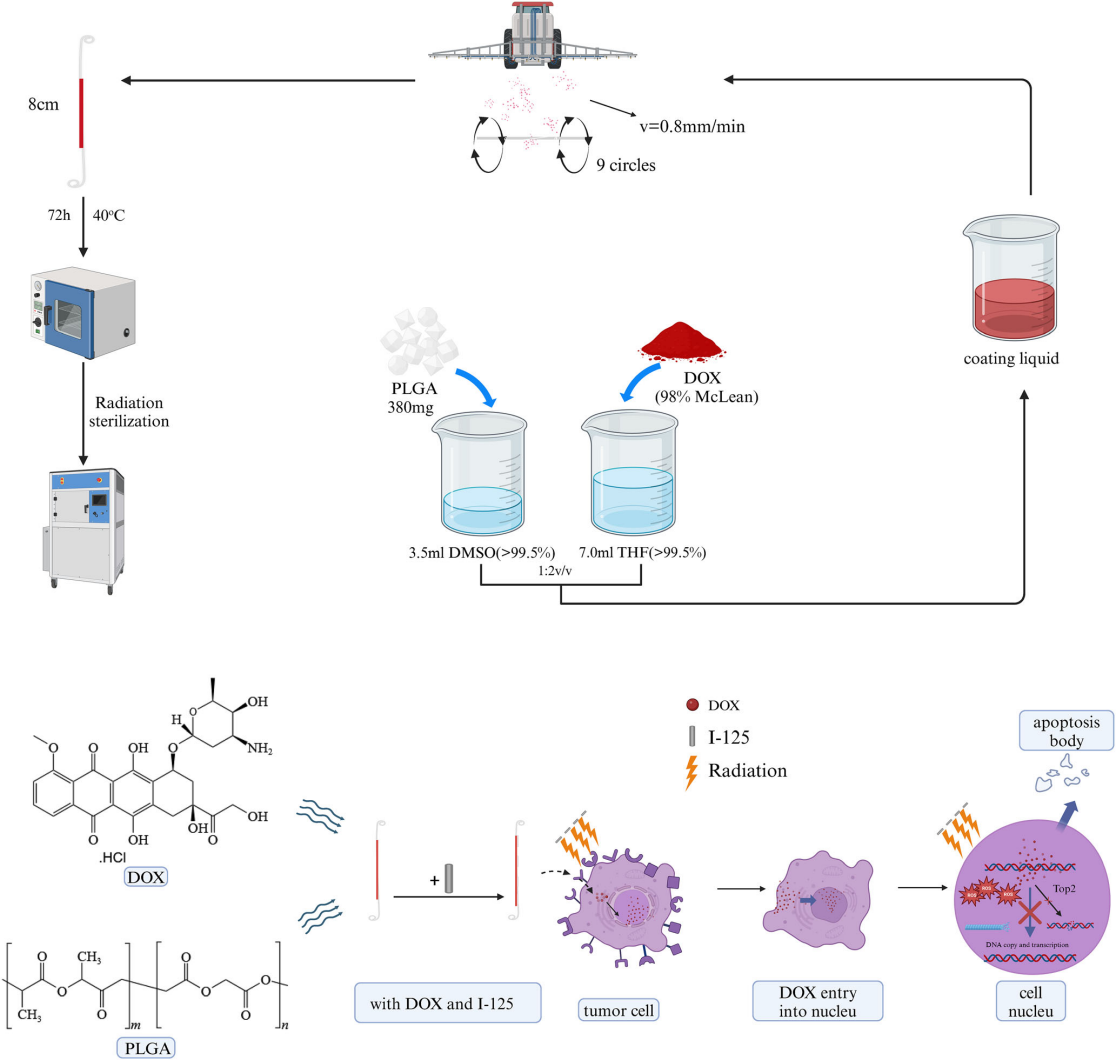
The fabrication process of IUS and its potential anticancer mechanism.

**FIGURE 2:**
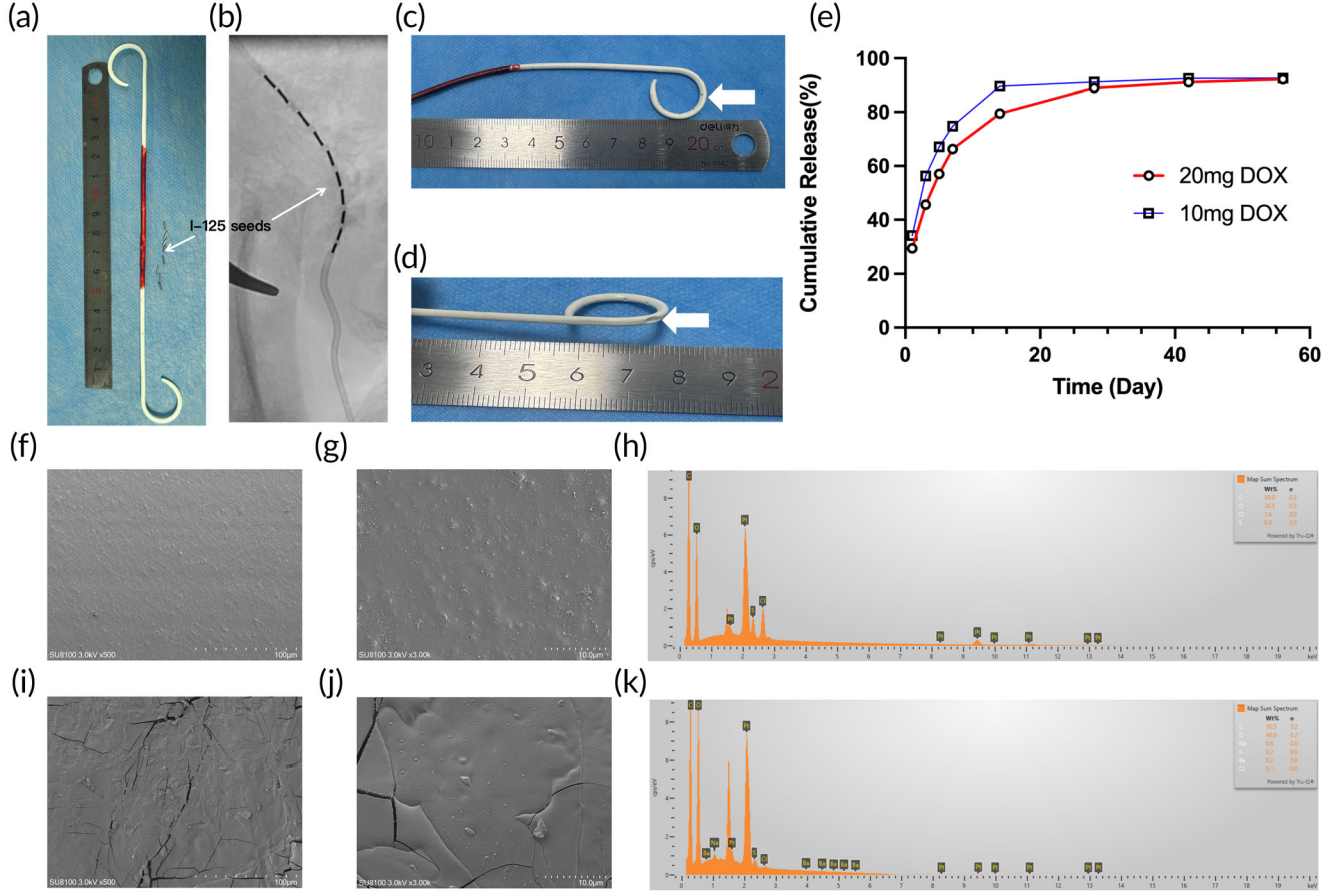
(a) The newly designed IUS features a dual‐cavity structure (a drainage cavity and ^125^I seed cavity) that is completely independent, with a DOX drug coating on the surface. (b) IUS implantation under DSA guidance. (c) The side holes at both IUS ends are used for urine drainage. (d) An opening hole 5 cm away from the head end serves as the seed implantation channel. (e) DOX release curve of IUS. (f–h) SEM imaging and energy‐dispersive spectroscopy (EDS) analysis of IUS. (i–k) SEM imaging and energy‐dispersive spectroscopy (EDS) analysis of the normal stent.

The design of the IUS is illustrated in Figure 2a–d. The stent was modified into a dual‐lumen structure, with one lumen accommodating 10 ^125^I seeds and the other serving as a drainage channel (Figure S3). Additionally, a DOX drug coating was applied to the surface of the IUS, as depicted in the schematic workflow in Figure 1. The drug‐loading capacity of the stent can be controlled by adjusting the concentration of the drug‐coating solution and the number of spray cycles; the detailed parameters are provided in Table S1. The in vitro drug release profile of the IUS is shown in Figure 2e, revealing initial burst release on the first day, followed by sustained release, with 90% of the drug released over 21 days. The morphology of the stent and the effects of the coating process were characterized by scanning electron microscopy (SEM) and energy‐dispersive spectroscopy (EDS). Specifically, Figure 2f–h displays the blank segment of the stent, while Figure 2i–k corresponds to the drug‐loaded segment. No significant cracks were observed on the stent surface, indicating minimal structural impact during the coating process. Although some areas exhibited uneven drug coating, the overall coating quality was satisfactory.

### 3.2 Design and characteristics of the PU‐DOX membrane

**FIGURE 3:**
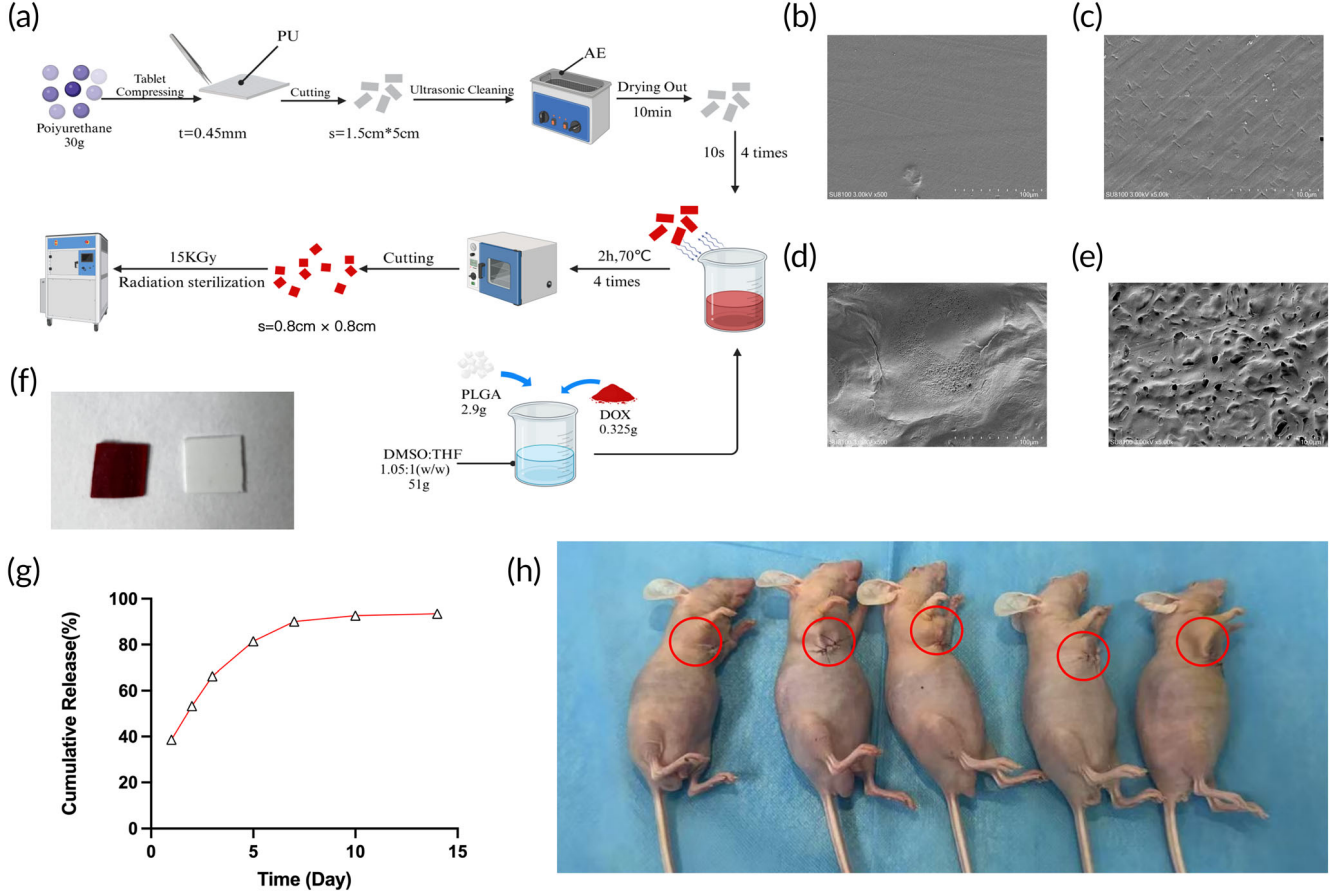
(a) Fabrication process of the DOX drug membrane. (b, c) SEM imaging of the blank membrane. (d, e) SEM imaging of the DOX drug membrane. (f) Photographs of the blank membrane and DOX drug membrane. (g) DOX release curve of the drug membrane. (h) Attachment of the drug membrane/blank membrane to the tumor surface in nude mice (red circle).

The design of the PU‐DOX membrane is illustrated in Figure 3a,f. Polyurethane, the same material used in the IUS, was selected as the drug delivery system for subsequent nude mouse experiments. Following electron beam sterilization, the original morphological structure of the polyurethane membrane was retained. The physical appearances and SEM images of the membranes are shown, with Figure 3b,c representing the non‐drug‐loaded membranes and Figure 3d,e the drug‐loaded membranes.

DOX was uniformly distributed across the surface of the polyurethane membrane, as confirmed by scanning electron microscopy, which demonstrated the homogeneous deposition of DOX on the membrane. The drug release profile of the membrane, also shown in Figure 3g, revealed initial burst release within the first hour, followed by gradual sustained release, with 90% of the drug released over more than 5 days. The relevant drug‐loading parameters are detailed in Table S2. The in vivo application of the membrane, which was surgically placed on the surface of nude mouse tumors, is depicted in Figure 3h, highlighting the practical implantation and its integration with the tumor site. All other mice with well‐adhered membranes remained in good health throughout the study, with no evidence of skin ulceration, local irritation, or systemic adverse effects, demonstrating the safety of the membrane.

### 3.3 in vitro cell experiments

#### 3.3.1 CCK8 assays

**FIGURE 4:**
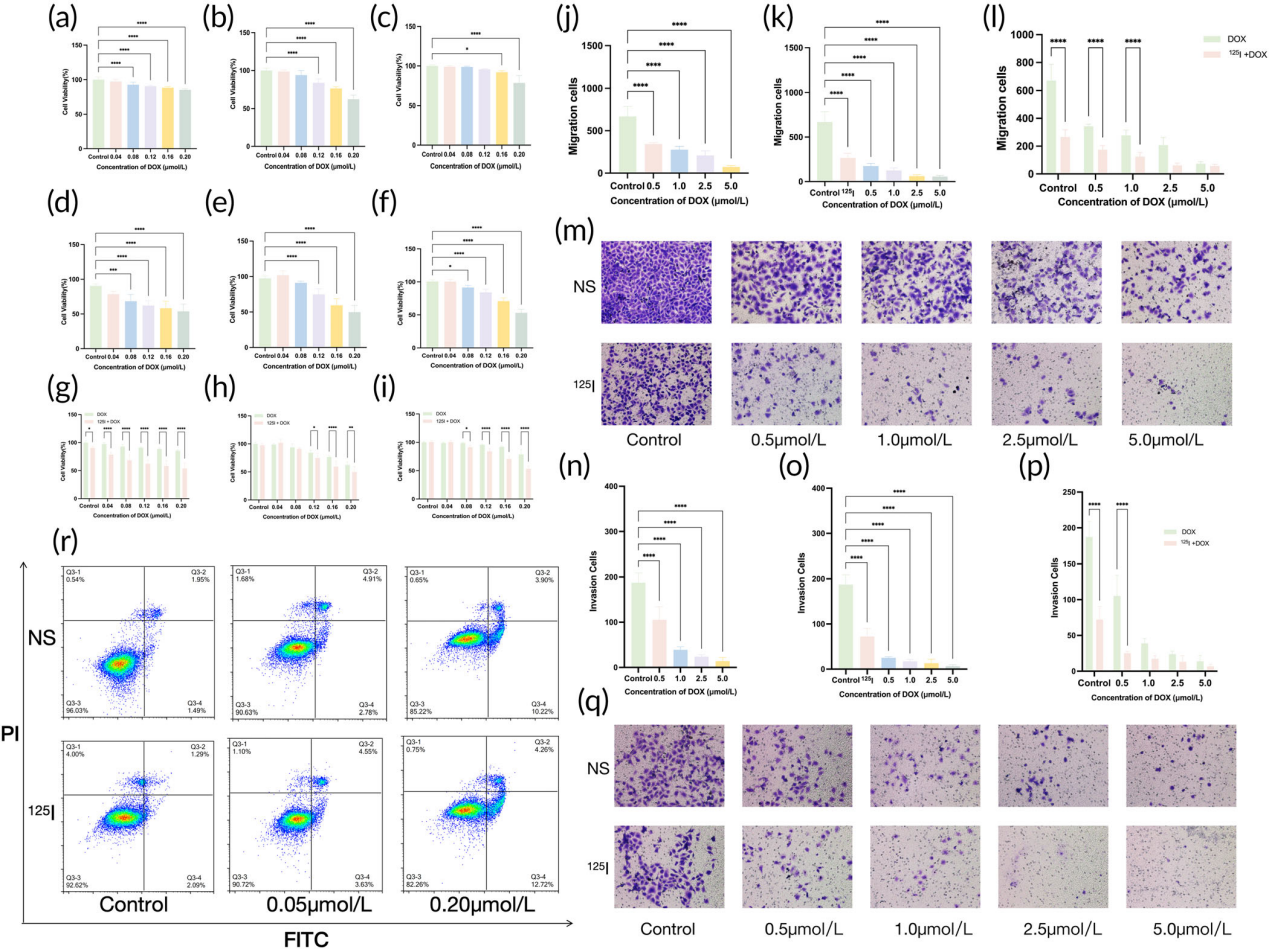
(a–c) CCK8 assay of normal cells treated with different concentrations of DOX for 24, 48, and 72 h. (d–f) CCK8 assay of ^125^I seed‐irradiated cells treated with the same DOX concentrations for 24, 48, and 72 h. (g–i) Comparison of cell viability between normal and ^125^I seed‐irradiated cells for 24, 48, and 72 h. (j–l) Transwell migration assays of normal and ^125^I seed‐irradiated cells under various conditions, with a comparative analysis. (m) Representative images of the Transwell migration assay. (n–p) Transwell invasion assays of normal and ^125^I seed‐irradiated cells under different conditions, with a comparative analysis. (q) Representative images of the Transwell invasion assay.

In the in vitro cell experiments, the viability of both normally cultured T24 cells and ^125^I‐irradiated T24 cells decreased significantly in a dose‐dependent manner with increasing concentrations of DOX (*p* < 0.01; Figure 4a,b,d,e). Notably, the reduction in cell viability was significantly greater in the ISB group than in the normally cultured group (*p* < 0.01; Figure 4c,f). This trend was consistently observed across all time points (24, 48, and 72 h), with the ^125^I‐irradiated cells exhibiting a more pronounced decline in viability at each interval relative to the controls (Figure 4g–i).

#### 3.3.2 Migration and invasion assays

Similarly, the migratory and invasive abilities of both normally cultured and ^125^I‐irradiated T24 cells decreased significantly in a dose‐dependent manner with increasing concentrations of DOX (*p* < 0.01; Figure 4j,k,n,o). Notably, the attenuations in migration and invasion were substantially more pronounced in the ^125^I‐irradiated cells than in the control cells across all the tested DOX concentrations (*p* < 0.01; Figure 4l,m,p,q).

#### 3.3.3 Apoptosis assay

As shown in this systematic evaluation of the proapoptotic effects of ^125^I and DOX on T24 cells, the apoptotic response of normally cultured T24 cells increased with increasing DOX concentration (0, 0.05, and 0.2 μmol/L), resulting in apoptosis rates of 3.97%, 9.37%, and 14.78%, respectively, including cells in both the early and late apoptotic phases (Figure 4r). In contrast, T24 cells treated with ^125^I exhibited significantly greater rates of apoptosis at the same DOX concentration, with rates of 7.38%, 9.28%, and 17.74%, respectively. The degree of apoptosis in ^125^I‐treated cells consistently exceeded that in normally cultured cells across all DOX concentrations, highlighting the enhanced proapoptotic efficacy of ^125^I combined with DOX.

### 3.4 In vivo BALB/c nude mouse experiments

#### 3.4.1 T24 tumor growth and volume in BALB/c‐nude mice

**FIGURE 5:**
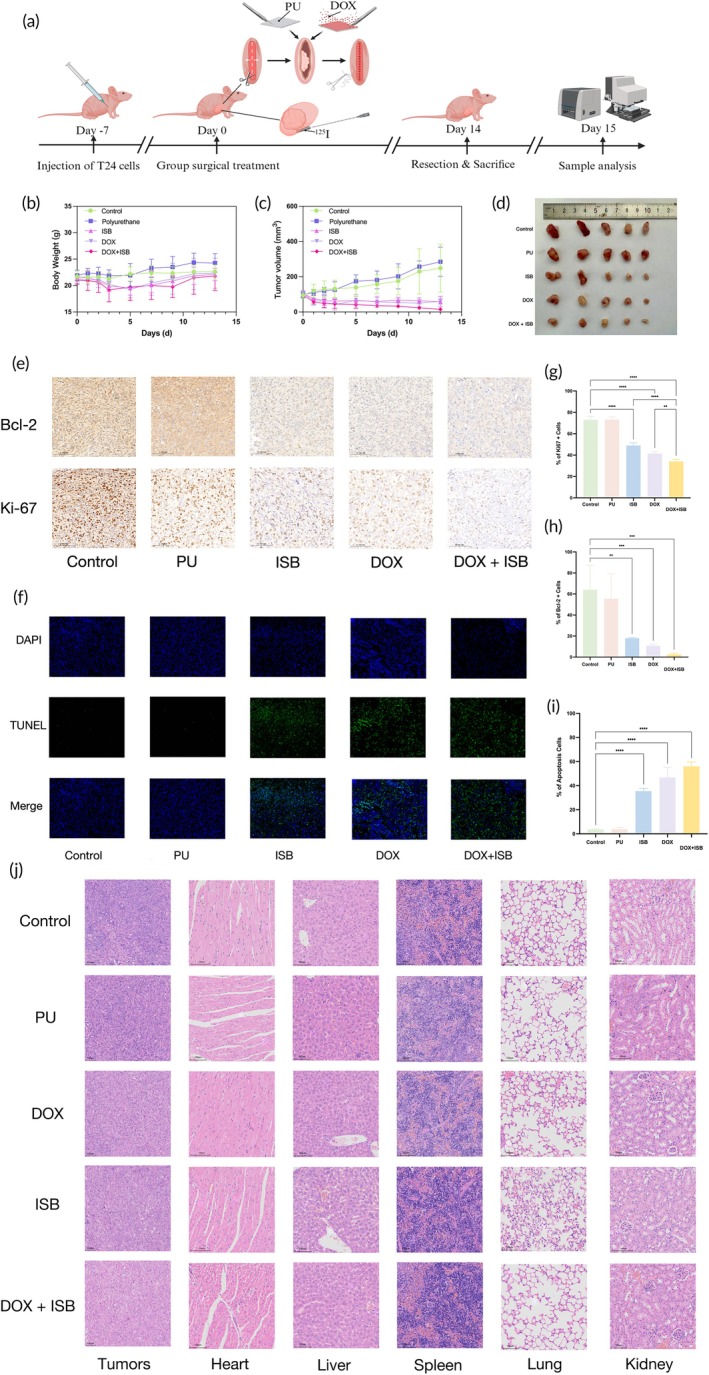
(a) Schematic of the procedure for the nude mouse experiment. (b, d) Tumor size and tumor volume growth curves for different groups of nude mice. (c) Body weight changes of nude mice. (e) Immunohistochemical staining for Bcl‐2 and Ki‐67 in nude mouse tumors. (f) TUNEL apoptosis assay in nude mouse tumors. (g–i) Positive rates of Ki‐67 and Bcl‐2, and apoptosis percentage in different groups. (j) HE staining of tumors, heart, liver, spleen, lungs, and kidneys. [Correction added on September 30, 2025, after first online publication: figure 5J was replaced by Addition of Scale Bars and Removal of Duplicated Images]

Following successful implantation of T24 tumors in BALB/c nude mice, and all nude mice survived after treatment, the body weights of the ISB, DOX, and (ISB + DOX) groups continuously decreased over the first 5 days, followed by gradual recovery. By Day 13, the body weights had returned to levels comparable to those in the Control and PU groups, with no statistically significant differences observed (Figure 5b). The tumor inhibition rate (IR) is presented in Table 1. Tumor growth and volume were assessed at multiple time points (Days 1, 2, 3, 5, 7, 9, 11, and 13) across the Control, PU, DOX, ISB, and (DOX + ISB) groups. Tumors in the Control and PU groups exhibited continuous growth, whereas tumors in the DOX, ISB, and (DOX + ISB) groups showed progressive reductions in size over time (Figure 5c,d). By Day 13, the tumor volumes in the DOX, ISB, and (DOX + ISB) groups were significantly smaller than those in the Control group (*p* < 0.0001), with no significant difference observed between the Control and PU groups (*p* = 0.618). Furthermore, the tumor volume in the (DOX + ISB) group was significantly smaller than that in the ISB group (*p* = 0.02), although no significant difference was found between the DOX + ISB and DOX groups (*p* = 0.09).

**TABLE 1:** Comparisons of IR.

Groups	Day 1	Day 2	Day 3	Day 5	Day 7	Day 9	Day 11	Day 13	*p*‐value (vs. Control)	*p*‐value (vs. DOX + ^125^I)
PU	7.50 ± 2.15	7.25 ± 2.33	3.65 ± 12.02	−25.14 ± 6.93	−15.26 ± 10.34	−15.14 ± 16.64	−11.96 ± 1.93	−14.57 ± 11.20	0.0613	<0.0001
DOX	43.97 ± 5.85	53.51 ± 6.83	69.00 ± 5.29	51.99 ± 5.24	63.83 ± 4.25	69.57 ± 1.66	78.12 ± 0.73	75.64 ± 0.41	<0.0001	0.0151
ISB	35.73 ± 0.67	50.44 ± 4.30	52.86 ± 0.88	53.95 ± 3.04	62.24 ± 2.19	61.62 ± 0.86	72.54 ± 0.56	76.40 ± 1.44	<0.0001	0.0891
DOX + ISB	51.57 ± 1.10	62.98 ± 3.62	64.82 ± 4.01	70.6 ± 2.12	77.05 ± 0.50	81.36 ± 0.07	89.77 ± 0.06	94.09 ± 0.01	<0.0001	NA

#### 3.4.2 HE staining

The HE staining results (Figure 5j) revealed that both ^125^I and DOX caused varying degrees of damage to tumor tissues compared with those in the Control group. Moreover, the combination of ISB and DOX resulted in significantly greater tumor damage. HE staining of the heart, liver, spleen, lungs, and kidneys revealed that the ISB and DOX drug wafers did not cause noticeable damage to these major organs.

#### 3.4.3 Immunohistochemistry

The expression levels of Ki‐67 and Bcl‐2 were significantly lower in the DOX, ISB, and (DOX + ISB) groups than in the Control and PU groups, with all differences reaching statistical significance (*p* < 0.05; Figure 5e,g,h). In contrast, no statistically significant differences were observed in the Ki‐67 and Bcl‐2 expression levels between the Control and PU groups (*p* > 0.05). Notably, the (DOX + ISB) group presented significantly greater Ki‐67 and Bcl‐2 expression levels than the DOX and ISB groups did (*p* < 0.05).

#### 3.4.4 TUNEL staining

The percentages of apoptotic cells were significantly greater in the DOX, ISB, and (DOX + ISB) groups than in the Control and PU groups (*p* < 0.05, Figure 5f,i). In contrast, no statistically significant difference in the number of apoptotic cells was detected between the Control and PU groups (*p* > 0.05). Furthermore, the percentage of apoptotic cells in the (DOX + ISB) group was markedly greater than that in the DOX and ISB groups (*p* < 0.05).

### 3.5 Beagle dog experiments

#### 3.5.1 Implantation of the IUS

**FIGURE 6:**
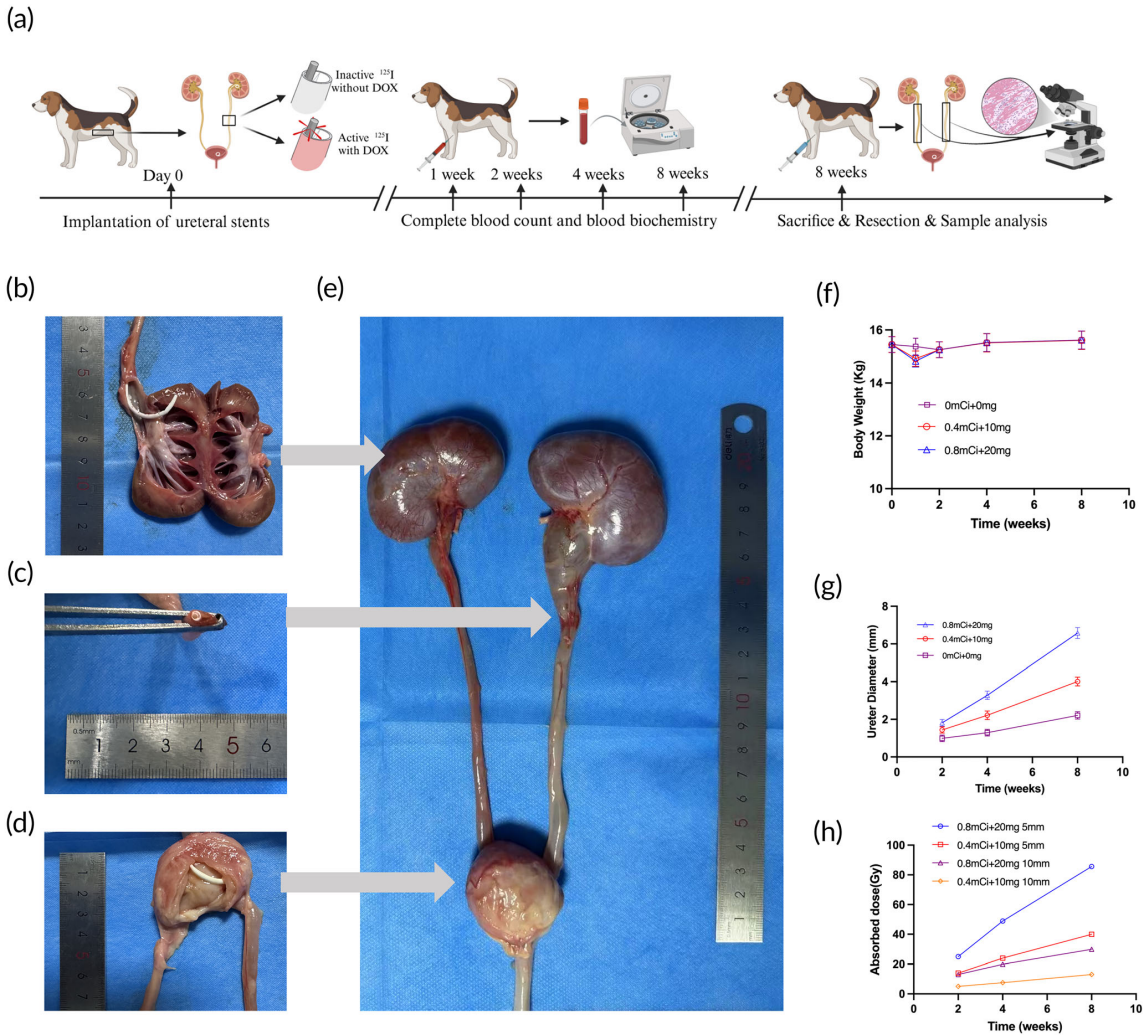
(a) Schematic of the Beagle dog experimental procedure. (b) Normal kidney color with expanded collecting system. (c) Cross‐section of the ureter after stent implantation. (d) The bladder portion of the BUS shows no secondary changes such as bladder mucosal hyperplasia. (e) Compared with the contralateral side, the kidneys and ureters of Beagle dogs show significant enlargement 8 weeks post‐surgery, with ureteral dilation and hydronephrosis. (f) Weight change curve of Beagle dogs. (g) Ureteral diameter changes at different time points for each group. (h) Changes in absorbed dose at each time point for each group.

All beagles successfully underwent IUS implantation without any surgery‐related mortality. Their body weight dynamic changes are shown in Figure 6f: a slight decrease was observed in the first week, followed by an increase to normal. All beagles remained in good health without signs of illness or surgery‐related complications.

#### 3.5.2 Serum biochemical examination

Following stent placement, the white blood cell (WBC) count in the (0.8 mCi + 20 mg) group increased significantly at 1 week (*p* < 0.05; Table 2) but returned to normal levels by weeks 2, 4, and 8. In contrast, the WBC counts in the (0.4 mCi + 10 mg) and (0 mCi + 0 mg) groups remained stable without significant changes throughout the observation period (Weeks 1, 2, 4, and 8).

**TABLE 2:** Blood test of 0.8 mCi + 20 mg animal group at different time.

Index (unit)	Baseline	1 week	2 week	4 week	8 week	Reference range
Red blood cells (10^6^/μL)	6.80 ± 0.50	6.90 ± 0.40	6.85 ± 0.32	6.30 ± 0.29	6.35 ± 0.51	5.57–7.82
White blood cell (10^3^/μL)	10.50 ± 2.05	13.30 ± 1.05^a^	13.06 ± 2.15^a^	10.76 ± 2.34	10.56 ± 0.85	5.81–14.71
Platelets (10^3^/μL)	272.21 ± 71.29	326.5 ± 20.16	297.6 ± 62.85	285.6 ± 35.60	290.5 ± 15.64	175.35–484.28
Hemoglobin (g/dL)	14.32 ± 1.44	15.04 ± 1.13	14.29 ± 1.05	14.00 ± 0.63	14.36 ± 0.65	11.95–17.68
Alanine aminotransferase (U/L)	28.7 ± 5.62	28.8 ± 5.57	30.7 ± 3.52	30.46 ± 6.46	30.71 ± 4.87	14.52–39.85
Aspartate aminotransferase (U/L)	36.5 ± 5.76	34.8 ± 4.08	36.6 ± 5.14	32.81 ± 3.15	39.24 ± 1.45	21.32–48.41
Total cholesterol (mmol/L)	4.25 ± 0.86	4.68 ± 0.64	4.58 ± 0.55	4.29 ± 0.43	4.55 ± 0.86	2.75–5.78
Triglyceride (mmol/L)	0.53 ± 0.12	0.56 ± 0.13	0.54 ± 0.14	0.60 ± 0.09	0.58 ± 0.15	0.54 ± 0.15
Total bilirubin (μmol/L)	1.55 ± 0.75	1.46 ± 0.45	1.43 ± 0.57	1.45 ± 0.74	1.63 ± 0.44	0.31–2.65
Direct bilirubin (μmol/L)	0.98 ± 0.52	1.05 ± 0.65	0.92 ± 0.48	0.95 ± 0.51	0.87 ± 0.41	0.01–1.85
Urea nitrogen (mmol/L)	4.26 ± 0.82	4.36 ± 1.07	4.73 ± 0.57	5.31 ± 0.09	5.81 ± 0.33	1.98–5.66
Creatinine (μmol/L)	40.9 ± 7.65	42.5 ± 7.28	43.2 ± 7.28	47.15 ± 4.00	49.12 ± 5.24	26.81–55.31
Blood glucose (mmol/L)	5.68 ± 0.43	5.85 ± 0.59	5.69 ± 0.51	5.85 ± 0.64	5.69 ± 0.80	4.87–6.81
Total protein (g/L)	56.8 ± 4.16	56.2 ± 3.84	55.7 ± 4.40	55.11 ± 5.18	55.14 ± 1.32	48.94–65.74
Albumin (g/L)	31.6 ± 3.21	31.5 ± 2.80	31.3 ± 2.14	30.82 ± 1.95	31.17 ± 0.71	27.63–37.75
Globulin (g/L)	24.8 ± 2.36	23.5 ± 2.46	23.6 ± 3.26	25.88 ± 1.58	25.77 ± 1.02	18.25–28.59

^a^ White blood cell (baseline vs. 1 week and baseline vs. 2 week) *p* < 0.05.

#### 3.5.3 Gross observations

All dogs undergoing IUS with 0.4 and 0.8 mCi seeds will do abdominal SPECT to detect precise γ‐ray coverage at 3 days after IUS placement (Figure S4). All ureters implanted with IUS exhibited varying degrees of hydronephrosis, which progressively worsened over time. However, no signs of hydronephrosis were observed in the contralateral ureters of any group (Figure 6b–e). The sections of IUS located at the upper or terminal pelvis (regions without seeds or DOX) showed no apparent damage. Detailed analysis of gross samples from the (0.8 mCi + 20 mg) group (Figure 6g) revealed gradual increases in ureteral diameter and wall thickness over time (Table 3), suggesting that radiation may induce chronic local tissue damage.

**TABLE 3:** Ureter diameter.

Group	Ureter diameter (mm)			*p*‐value
	2 weeks	4 weeks	8 weeks	
0 mCi + 0 mg group	1.0 ± 0.10	1.3 ± 0.15	2.2 ± 0.20	*p <* 0.0001
0.4 mCi + 10 mg	1.5 ± 0.12	2.2 ± 0.2	4.0 ± 0.25	*p <* 0.0001
0.8 mCi + 20 mg	1.8 ± 0.15	3.2 ± 0.25	6.5 ± 0.30	*p <* 0.0001

#### 3.5.4 HE and Sirius red staining

**FIGURE 7:**
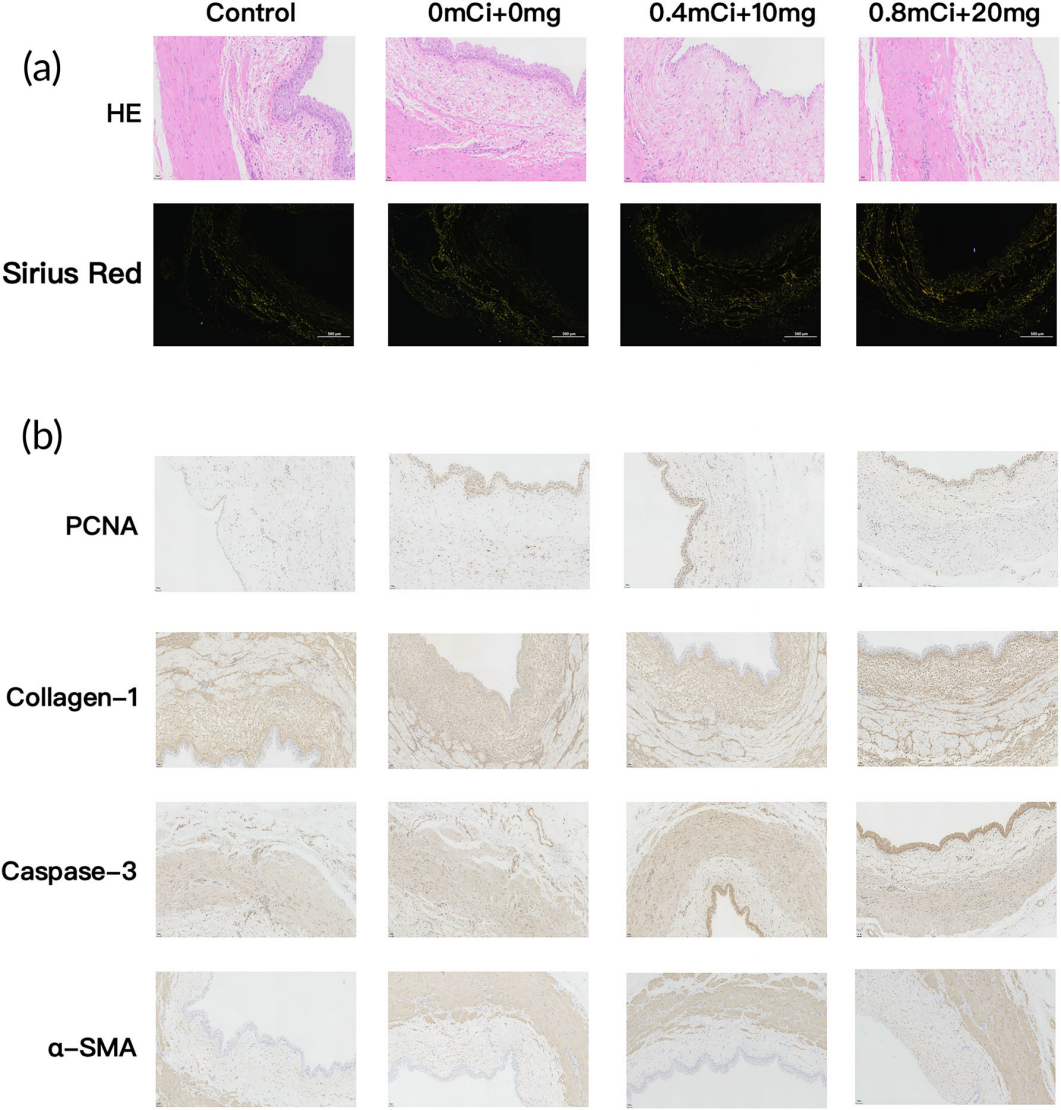
(a) HE and Sirius red staining of different groups after 8 weeks. (b) Immunohistochemical staining of α‐SMA, PCNA, caspase‐3, and Collagen‐I in different groups after 8 weeks. [Correction added on September 30, 2025, after first online publication: Figure 7B is replaced with correct Image Positions]

At the 8‐week time point, HE staining revealed varying degrees of damage to the transitional epithelium of the ureteral wall, with the most severe damage observed in the (0.8 mCi + 20 mg) group. Sirius red staining revealed varying degrees of fibrosis across the different groups, with the most pronounced fibrosis observed in the (0.8 mCi + 20 mg) group (Figure 7a).

#### 3.5.5 Radiation dosimetry

Table 4 summarizes the absorbed radiation doses in adjacent tissues at reference points 5 and 10 mm from the BUS (Figure 6h) on the brachytherapy treatment system. At 8 weeks, the cumulative dose in the 0.8 mCi group was the highest, reaching 84.29 Gy.

**TABLE 4:** Absorbed radioactive dosages of the tissues adjacent to the stent at a distance of 5 and 10 mm from IUS.

Group	Absorbed dosage (5 mm from IUS, Gy)			Absorbed dosage (10 mm from IUS, Gy)		
	2 weeks	4 weeks	8 weeks	2 weeks	4 weeks	8 weeks
0.4 mCi + 10 mg	14.07	25.24	41.78	5.40	9.38	15.92
0.8 mCi + 20 mg	25.66	49.41	84.29	10.33	18.96	33.43

#### 3.5.6 Immunohistochemistry

Compared with those in the Control group, the protein expression levels of PCNA, α‐SMA, collagen I, and caspase‐3 in the treatment groups significantly increased in a time‐dependent manner. At each time point, PCNA expression was lowest in the (0.8 mCi + 20 mg) group, with the lowest level observed at 8 weeks. In contrast, the α‐SMA, collagen I, and caspase‐3 expression levels were highest in the (0.8 mCi + 20 mg) group and lowest in the (0 mCi + 0 mg) group (Figure 7b).

#### 3.5.7 Immunofluorescence staining

Immunofluorescence staining was performed to analyze the expression levels of key fibrotic, proliferative, and apoptotic markers, including PCNA, caspase‐3, collagen I, and α‐SMA. These results were consistent with the immunohistochemistry findings.

**FIGURE 8:**
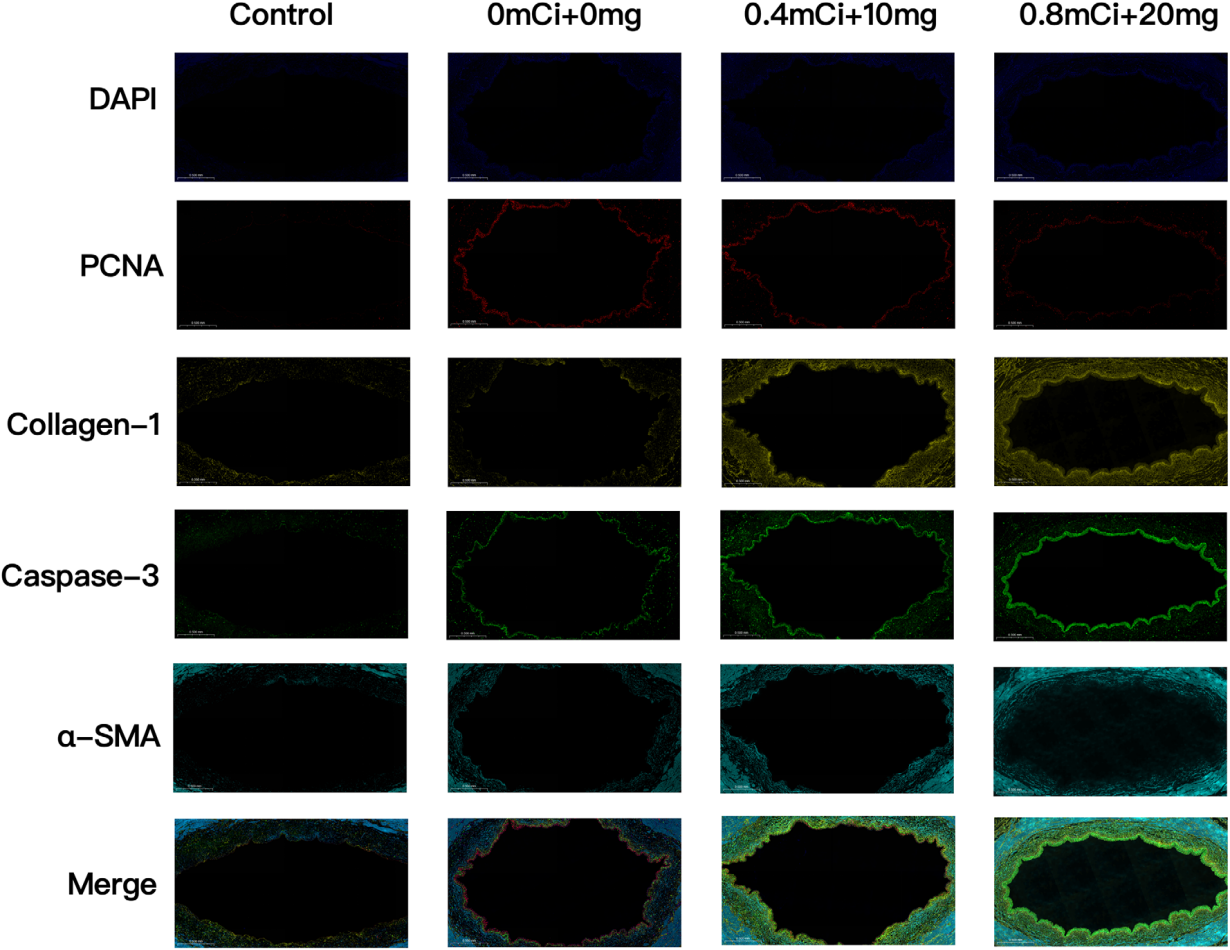
Immunofluorescence staining of α‐SMA, PCNA, caspase‐3, and Collagen‐I in different groups after 8 weeks.

At 8 weeks, significant differences in the expression levels of these markers were observed between the control and treatment groups. At each time point, PCNA expression was lowest in the 0.8 mCi group, with the lowest level observed at 8 weeks. In contrast, the expression levels of α‐SMA, collagen I, and caspase‐3 were highest in the 0.8 mCi group and lowest in the 0 mCi group at 2, 4, and 8 weeks, respectively (Figure 8).

## 4 DISCUSSION

Radical nephroureterectomy remains the gold standard treatment for UC.^12^, ^13^ The rationale for this aggressive surgical approach lies in its ability to achieve complete oncological control by eliminating the primary tumor and potential sites of microscopic disease spread.^14^ However, surgery has several drawbacks, including perioperative morbidity, reduced quality of life, and long‐term metabolic complications, particularly in vulnerable populations.^15^ These limitations underscore the urgent need for less invasive, nephron‐sparing approaches and the development of effective systemic therapies.

ISB has been extensively utilized in the treatment of various malignancies and has demonstrated significant efficacy and several advantages.^16^, ^17^ Jiao^18^ first evaluated the ISB for UC, and technical success was 100% without major complications, local tumor progression was absent, and the median overall survival was 25 months. DOX is commonly used for treating urinary system neoplasms, with intravesical and intravenous routes offering distinct benefits and risks. Intravesical administration involves direct bladder instillation, minimizing systemic exposure. A study demonstrated that a single immediate postoperative intravesical instillation of DOX significantly reduced the risk of recurrence in patients with nonmuscle‐invasive bladder cancer, providing an effective and cost‐efficient alternative to other chemotherapeutic agents.^19^ In contrast, intravenous DOX is associated with systemic exposure and cardiotoxicity. The combination of ISB and DOX may offer potential for more effective urinary cancer treatment. ISB provides localized radiotherapy, and locally delivered DOX enhances chemotherapy effects, potentially reducing recurrence and minimizing cardiotoxicity.

We evaluated the effects of DOX and ISB on T24 cell proliferation, migration, invasion, and apoptosis. The results revealed that increasing the DOX concentration reduced cell proliferation, migration, and invasion while increasing apoptosis, which is consistent with previous findings.^20^ Notably, ISB significantly enhanced these effects, indicating a synergistic anticancer effect when combined with DOX. Although ISB alone did not always produce significant results in some assays, this could be due to insufficient ISB time, as suggested by earlier studies.^21^, ^22^ In conclusion, our in vitro data support the potential of ISB combined with DOX as an effective therapeutic strategy for UC.

A xenograft tumor study showed no significant difference in tumor size between the Control and PU groups, confirming that the PU membrane did not affect tumor growth. In contrast, the ISB, DOX, and DOX + ISB groups presented significantly smaller tumors, with the combination therapy resulting in the smallest tumors, indicating a synergistic effect. These findings align with prior studies demonstrating the enhanced efficacy of combining ISB with chemotherapy.^23^ Body weights remained stable in the Control and PU groups, whereas the ISB, DOX, and DOX + ISB groups experienced temporary weight loss followed by recovery, suggesting good treatment tolerability.

HE staining revealed no significant differences in cellular atypia or chromatin coarseness between the Control and PU groups; however, these features were progressively reduced in the ISB, DOX, and especially the DOX + ISB groups, indicating improved tumor cell differentiation and reduced malignancy. Immunohistochemical analysis revealed that Ki‐67 and Bcl‐2 expression levels were significantly lower in the ISB, DOX, and DOX + ISB groups than in the control and PU groups, with the combination therapy (DOX + ISB) resulting in the greatest reduction. TUNEL assay results revealed increased apoptosis in the treated groups, with the highest levels in the (DOX + ISB) group. These results support the potential of this combination therapy as an effective cancer treatment, corroborating prior research.^24^

All beagle dogs in the implantation groups survived. Despite temporary weight loss in the first week and a transient WBC increase in the first 2 weeks, both returned to normal. Most dogs resumed normal activity the day after IUS implantation, indicating that IUS is safe and has minimal systemic effects even with a maximum of 84.29 Gy. Although the effects of ISB injuries to nerves, blood vessels, and the trachea have been reported in the past, all the results revealed slight or no damage to the target or surrounding organs.^25^, ^26^ Moreover, even with the addition of DOX, IUS remains highly safe, as DOX can achieve high local perfusion concentrations—up to 1.25 mg/mL in humans^27^ and 0.4 mg/mL in nude mice^28^ without causing severe tissue damage. Pathological analysis indicated that implantation duration, DOX dosage, and ^125^I activity were key factors in ureteral fibrosis, injury, and expansion. HE staining revealed varying degrees of transitional epithelium damage with increasing drug release and radiation doses, whereas Sirius red staining confirmed fibrosis as collagen fiber proliferation. These findings support IUS as a feasible localized treatment, emphasizing the need to optimize the dosage and implantation time.

**FIGURE 9:**
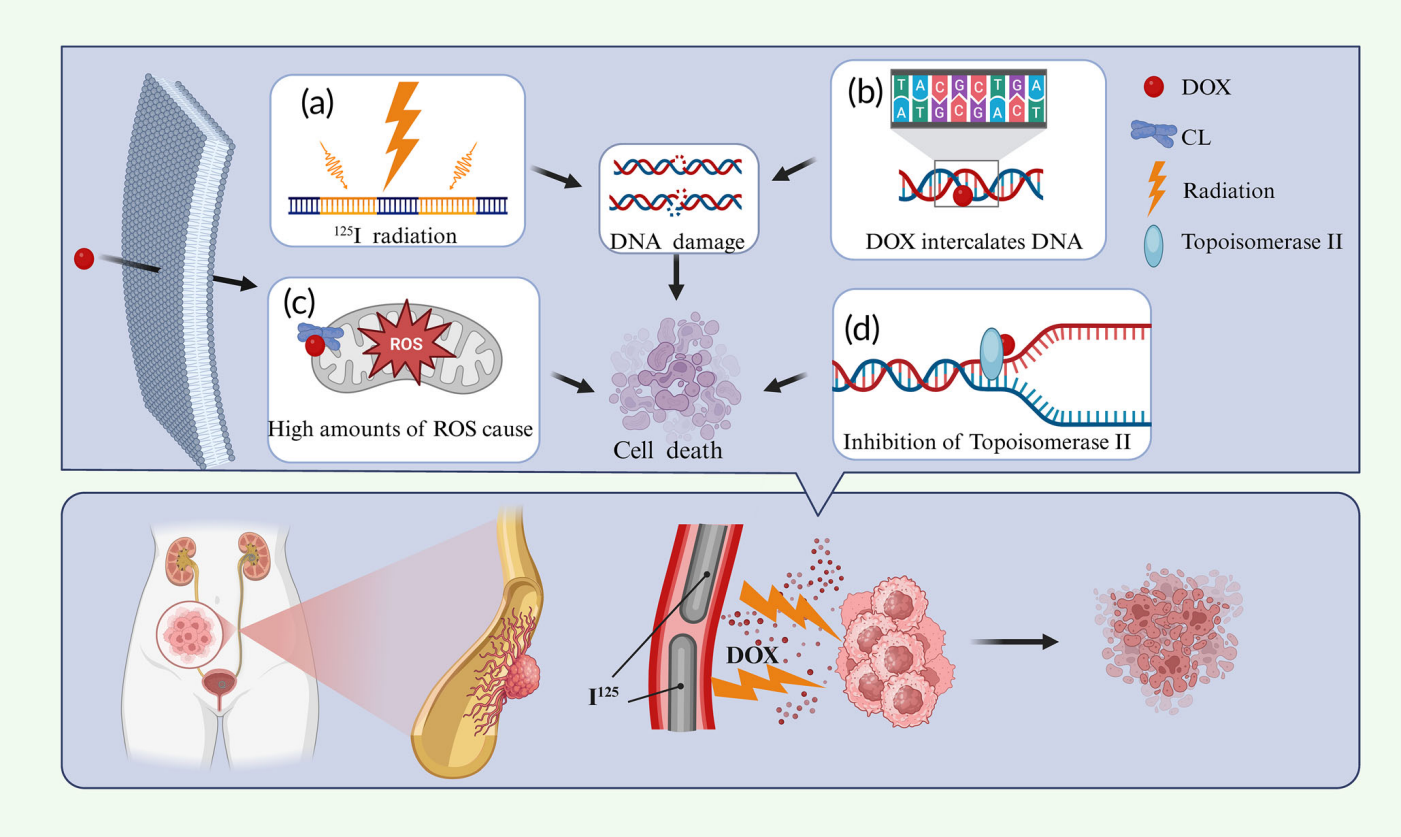
Possible anticancer mechanisms of ^125^I seeds and doxorubicin. (a, b) DNA damage. (c) Cause of ROX. (d) Inhibition of Topoisomerase II.

ISB applies the “4R” concept (DNA repair, ROX generation, repopulation, and cell cycle redistribution) with no tumor cell repopulation, minimal cell cycle redistribution, and a low oxygen enhancement ratio.^29^ The radiobiological mechanisms of ISB remain poorly understood, with most research focusing on the dose rate effect. Radiation‐induced cell death involves potentially lethal damage and sublethal damage that can accumulate during fractionated irradiation. At brachytherapy dose rates ranging from 0.3 Gy/h to 1 Gy/min, DNA repair dominates cell lethality.^30^ The ISB dose rate depends on seed radioactivity, with 1 Gy/h resulting in effects similar to those of 2 Gy fractions in conventional radiotherapy.^31^ Tumor radiosensitivity varies by genotype.^32^ Precise dose delivery is crucial, but the immunological effects of ISB remain unclear.^33^ DOX intercalates into DNA, untwisting and supercoiling it, and binds cardiolipin in mitochondria, generating ROS and triggering apoptosis.^34^ In the nucleus, DOX inhibits topoisomerase II at concentrations >0.25 μg/mL, leading to DNA cleavage and apoptosis (Figure 9a–d).^35^

One major limitation of this study is the difficulty in establishing an in situ UC model, making it challenging to directly verify the effects of DOX and ISB on UC. Additionally, the smaller ureteral diameter in dogs than in humans poses another challenge. The newly designed IUS has a relatively small diameter, which does not fully replicate human ureteral injury. The mismatch between the IUS and dog ureters increases the risk of mechanical injury, limiting its translational potential. Future improvements could include the exploration of different drug‐loading strategies, such as gemcitabine, docetaxel, or immunotherapeutic agents, to increase therapeutic efficacy. Additionally, the use of radiotherapy sensitizers could further improve treatment outcomes while maintaining safety.

## 5 CONCLUSIONS

We developed a novel IUS with integrated chemo‐radiotherapy functionality. The combination of ISB and DOX has potent antitumor effects. Compared with traditional double‐J stents, this innovative stent not only ensures effective urinary drainage but also delivers targeted tumor treatment. Clinical studies will be required to test its use in humans.

## AUTHOR CONTRIBUTIONS

**Xiaotian Yang and Xueliang Zhou**: Conceptualization; data curation; formal analysis; writing – original draft; writing – review and editing. **Zhanyun Zhou**: Data curation; investigation; software; writing – original draft. **Yipu Li**: Investigation; resources. **Chengzhi Zhang**: Resources; supervision; validation. **Yingqi Liu, Xiaohan Ma, Yanan Li, and Yebin Wang**: Investigation; resources; validation. **Dechao Jiao**: Conceptualization; data curation; formal analysis; investigation; writing – review and editing.

## CONFLICT OF INTEREST STATEMENT

The authors declare no conflicts of interest.

## Supporting information


**Figure S1.** Standard calibration curve of doxorubicin (DOX) in PBS measured at 485 nm.
**Figure S2.** Ex vivo ^125^I seed‐based brachytherapy model for in vitro cell studies (a) front view; (b) top view; (c) brachytherapy model used in the Transwell assay.
**Figure S3.** (a) Surface view of ureteral stent; (b) longitudinal section of ureteral stent.
**Figure S3.** ^125^I irradiation distribution showed good SPECT at 3 days after operation.
**Figure S4.** SPECT 3 days after IUS placement, Gamma‐rays completely cover the ureter of dogs after postoperation on SPECT (arrow).
**Figure S5.** The drug‐coating process of the stent was performed using an ultrasonic spraying device (MediCoat BCC‐300, SonoTek Corporation, Milton, NY).
**Table S1.** Property parameters of DOX stent coatings.
**Table S2.** Property parameters of DOX drug membrane coatings.

## Data Availability

The data that support the findings of this study are available from the corresponding author upon reasonable request.
