# Supplementary material for: A novel brachytherapy and chemotherapy integrated ureteral stent: In vitro and in vivo study
Source: Bioeng Transl Med. 2025 Sep 19;10(6):e70077. doi: 10.1002/btm2.70077 (PMC12617561; doi:10.1002/btm2.70077)
Supplement: Supplementary file 1 — Figure S1. Standard calibration curve of doxorubicin (DOX) in PBS measured at 485 nm. Figure S2. Ex vivo 125I seed‐based brachytherapy model for in vitro cell studies (a) front view; (b) top view; (c) brachytherapy model used in the Transwell assay. Figure S3. (a) Surface view of ureteral stent; (b) longitudinal section of ureteral stent. Figure S3. 125I irradiation distribution showed good SPECT at 3 days after operation. Figure S4. SPECT 3 days after IUS placement, Gamma‐rays completely cover the ureter of dogs after postoperation on SPECT (arrow). Figure S5. The drug‐coating process of the stent was performed using an ultrasonic spraying device (MediCoat BCC‐300, SonoTek Corporation, Milton, NY). Table S1. Property parameters of DOX stent coatings. Table S2. Property parameters of DOX drug membrane coatings. [file BTM2-10-e70077-s001.docx]

**Supplementary Materials**

**Supplementary Figure S1** Standard calibration curve of doxorubicin (DOX) in PBS measured at 485 nm


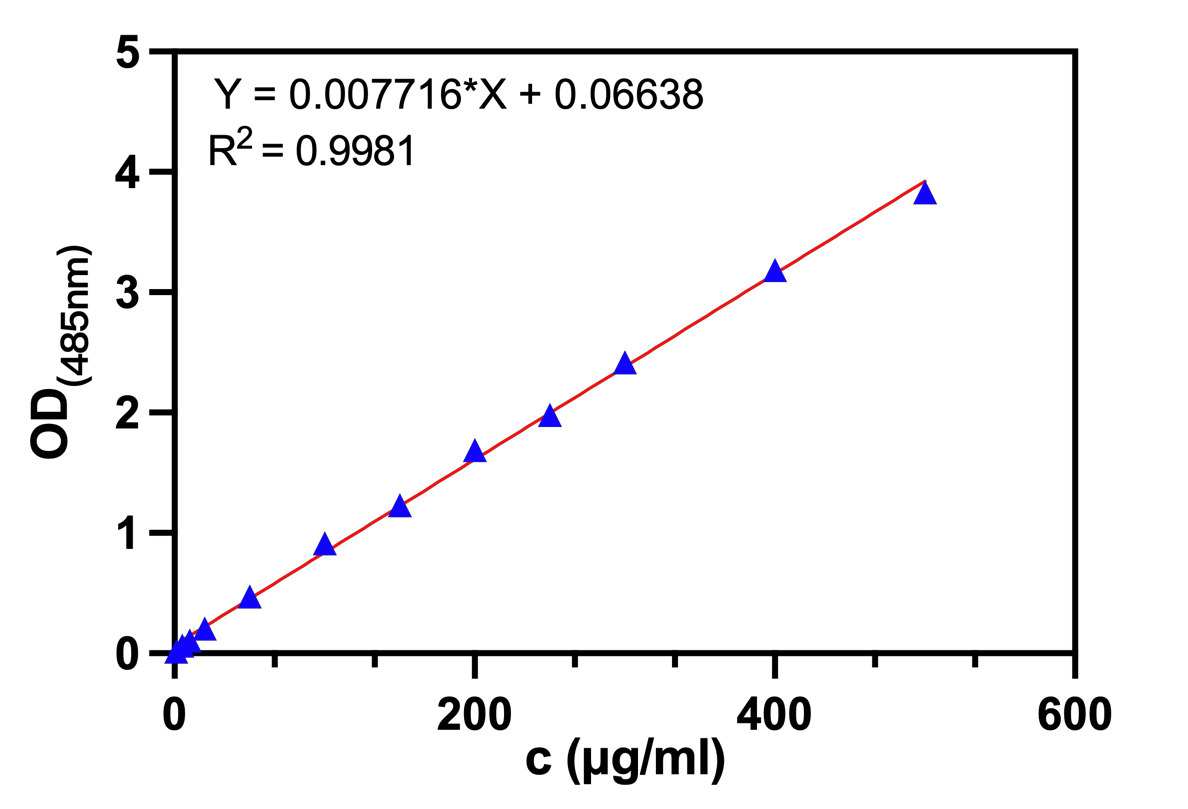


**Supplementary Figure S2** Ex vivo ^125^I seed-based brachytherapy model for in vitro cell studies (a) Front view; (b) Top view; (c) brachytherapy model used in the Transwell assay


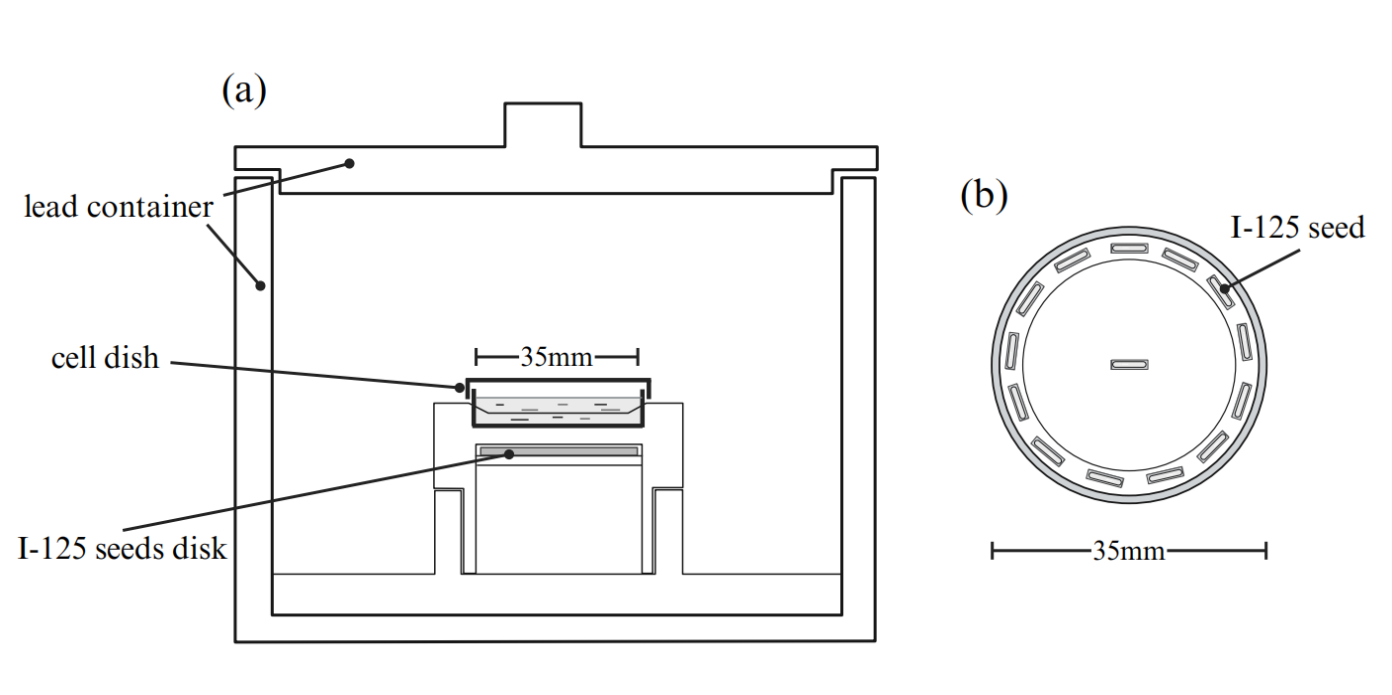


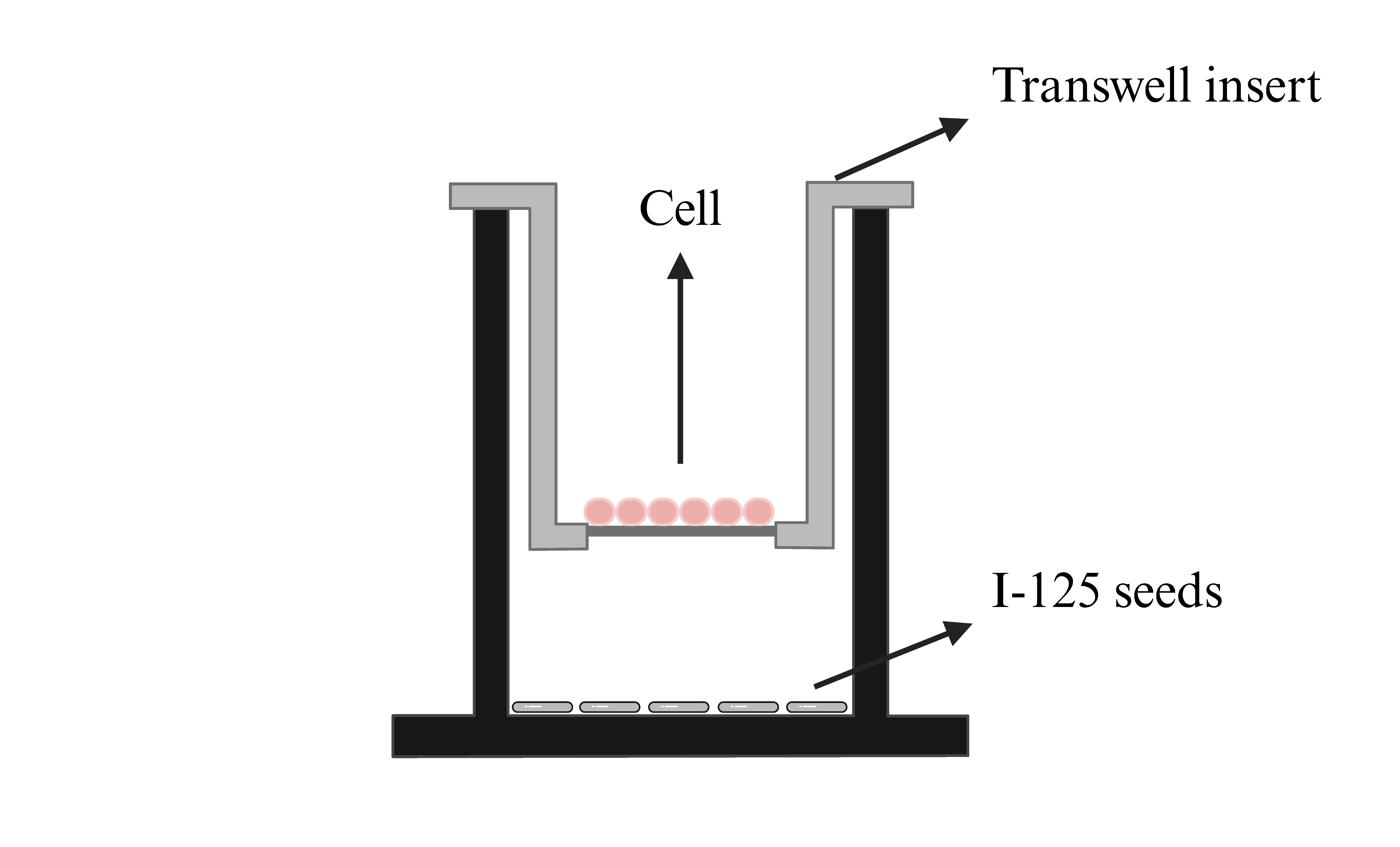


(c)

**Supplementary Figure S3** (a) Surface view of ureteral stent; (b)Longitudinal section of ureteral stent

**

**

**Supplementary Figure S3** ^125^I irradiation distribution showed good SPECT at 3 days after operation

**Supplementary Figure S4** SPECT 3 days after IUS placement, Gamma-rays completely cover the ureter of dogs after postoperation on SPECT (arrow)


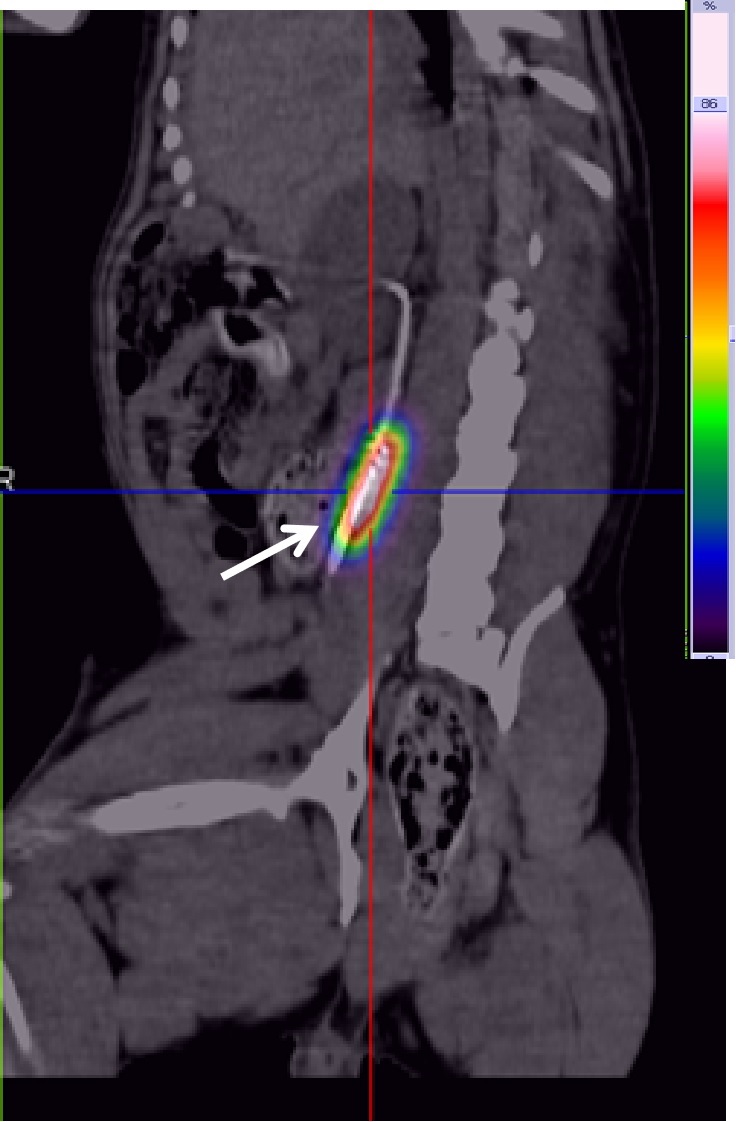


**Supplementary Figure S5** The drug-coating process of the stent was performed using an ultrasonic spraying device (MediCoat BCC-300, SonoTek Corporation, Milton, NY, USA).

**
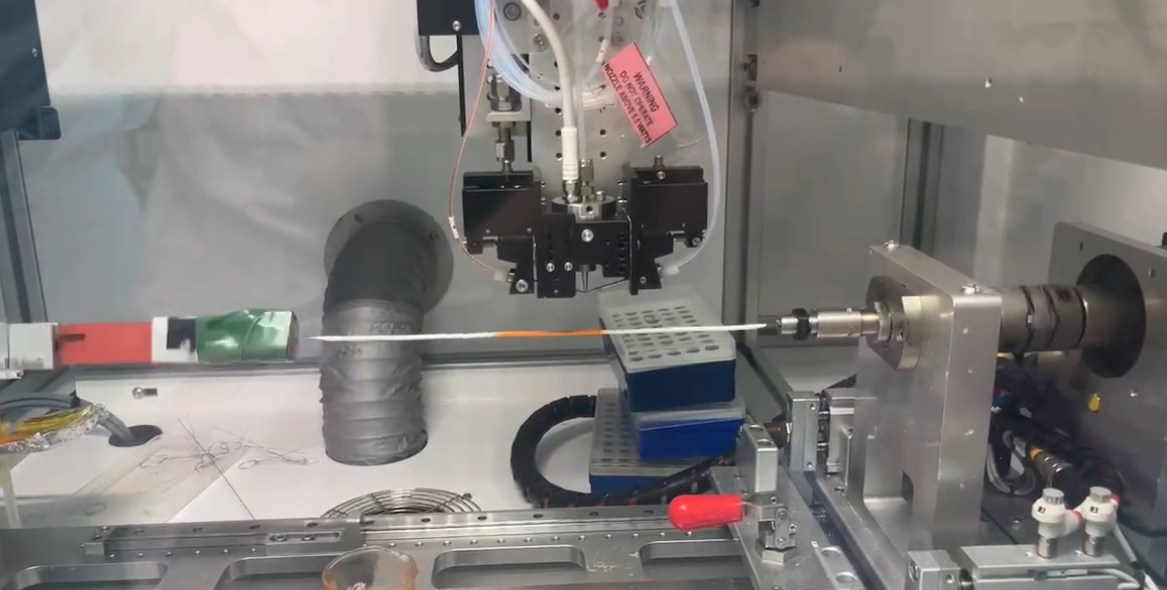
**

**Supplementary Table S1** Property parameters of DOX stent coatings

| Group | Spray coating liquid | | spraying/circles | DOX loading capacity* | 90% DOX Release |
| --- | --- | --- | --- | --- | --- |
|  | DOX/PLGA | THF/DMSO |  |  |  |
| DOX-High | 2.3 | 2/1 | 5-6 | 19.45±0.78 mg | 29.4±4.3 d |
|  | 1 |  | 8-10 | 18.44±0.64mg | 38.6±3.7 d |
| DOX-Low | 2.3 |  | 3-4 | 10.12±0.43mg | 14.3±2.4 d |
|  | 1 |  | 5-6 | 9.44±0.21mg | 18.7±2.1 d |

“*”: The drug loading was calculated based on the weight difference before and after coating, multiplied by the drug percentage in the extraction solution

**Supplementary Table S2** Property parameters of DOX drug membrane coatings

| Parameters of the extraction solution | | DOX loading capacity* |
| --- | --- | --- |
| Type | content /mg |  |
| PLGA | 2900 | 90.24±23.44/ cm^2^ |
| DOX-HCl | 325 |  |
| THF/DMSO | 1/1.05 |  |

“*”Drug loading was determined based on the drug release profile in PBS.
